# Atrophy Rates in Asymptomatic Amyloidosis: Implications for Alzheimer Prevention Trials

**DOI:** 10.1371/journal.pone.0058816

**Published:** 2013-03-15

**Authors:** K. Abigail Andrews, Marc Modat, Kate E. Macdonald, Tom Yeatman, M. Jorge Cardoso, Kelvin K. Leung, Josephine Barnes, Victor L. Villemagne, Christopher C. Rowe, Nick C. Fox, Sebastien Ourselin, Jonathan M. Schott, the Australian Imaging Biomarkers, Lifestyle Flagship Study of Ageing

**Affiliations:** 1 Dementia Research Centre (DRC), University College London, London, United Kingdom; 2 Centre for Medical Image Computing (CMIC), University College London, London, United Kingdom; 3 Austin Health, Department of Nuclear Medicine and Centre for PET, Heidelberg, Victoria, Australia; Nathan Kline Institute and New York University School of Medicine, United States of America

## Abstract

There is considerable interest in designing therapeutic studies of individuals at risk of Alzheimer disease (AD) to prevent the onset of symptoms. Cortical β-amyloid plaques, the first stage of AD pathology, can be detected *in vivo* using positron emission tomography (PET), and several studies have shown that ∼1/3 of healthy elderly have significant β-amyloid deposition. Here we assessed whether asymptomatic amyloid-PET-positive controls have increased rates of brain atrophy, which could be harnessed as an outcome measure for AD prevention trials. We assessed 66 control subjects (age = 73.5±7.3 yrs; MMSE = 29±1.3) from the Australian Imaging Biomarkers & Lifestyle study who had a baseline Pittsburgh Compound B (PiB) PET scan and two 3T MRI scans ∼18-months apart. We calculated PET standard uptake value ratios (SUVR), and classified individuals as amyloid-positive/negative. Baseline and 18-month MRI scans were registered, and brain, hippocampal, and ventricular volumes and annualized volume changes calculated. Increasing baseline PiB-PET measures of β-amyloid load correlated with hippocampal atrophy rate independent of age (p = 0.014). Twenty-two (1/3) were PiB-positive (SUVR>1.40), the remaining 44 PiB-negative (SUVR≤1.31). Compared to PiB-negatives, PiB-positive individuals were older (76.8±7.5 vs. 71.7±7.5, p<0.05) and more were *APOE4* positive (63.6% vs. 19.2%, p<0.01) but there were no differences in baseline brain, ventricle or hippocampal volumes, either with or without correction for total intracranial volume, once age and gender were accounted for. The PiB-positive group had greater total hippocampal loss (0.06±0.08 vs. 0.02±0.05 ml/yr, p = 0.02), independent of age and gender, with non-significantly higher rates of whole brain (7.1±9.4 vs. 4.7±5.5 ml/yr) and ventricular (2.0±3.0 vs. 1.1±1.0 ml/yr) change. Based on the observed effect size, recruiting 384 (95%CI 195–1080) amyloid-positive subjects/arm will provide 80% power to detect 25% absolute slowing of hippocampal atrophy rate in an 18-month treatment trial. We conclude that hippocampal atrophy may be a feasible outcome measure for secondary prevention studies in asymptomatic amyloidosis.

## Introduction

Despite intensive efforts, pharmacological strategies aimed at disrupting the underlying pathology of Alzheimer’s disease (AD) have thus far failed to translate into therapies for patients. One explanation is that trials are being carried out too late in the disease, and there is therefore considerable interest in identifying and treating individuals as early as possible, with the aim of slowing or ideally preventing the development of cognitive symptoms [1]. The development of biomarkers has made early AD detection realistic: amyloid pathology can be quantified using positron emission tomography (PET) [2], [3]; Aβ1-42 and tau can be measured in cerebrospinal fluid (CSF) [4]; and rates of cerebral atrophy can be quantified from serial MRI [5]. Numerous biomarker studies have produced evidence that the AD pathological cascade starts many years prior to symptoms [6], paving the way both for pre-symptomatic diagnosis of AD [7], and studies aimed at preventing or delaying the onset of symptoms - secondary prevention [1], [8]. Recent CSF [9] and amyloid PET [10]–[13] studies have shown that around a third of cognitively normal individuals in their 70 s have significant amyloid deposition. Whilst it is not yet known whether all individuals with “asymptomatic amyloidosis” will develop AD if they live long enough, on a group level some [14], if not all [15] studies have shown that these individuals have worse cognition, increased cognitive decline [16], [17], with some early evidence for a higher risk of subsequent conversion to MCI or AD [16], [18]. Major therapeutic studies targeting elderly individuals with asymptomatic amyloidosis are currently planned [19]. Here, we aimed to assess whether using amyloid PET as an inclusion criteria, and atrophy measured from serial MRI as an outcome measure, might be a feasible means of helping to determine disease modifying effects in AD secondary prevention studies.

## Materials and Methods

### Subjects

We used data from the Australian Imaging Biomarkers and Lifestyle (AIBL) study. We included subjects designated as healthy controls at baseline who had a Pittsburgh Compound B (PiB) ^11^C PET scan and two 3T MRI volumetric scans ∼18 months apart. Details of the AIBL methodology have previously been reported [20]. In brief healthy controls ≥60 years old were recruited from the community, with ∼50% having subjective memory complaints and ∼50% being *APOE4* carriers. All subjects underwent standardised clinical and neuropsychological examinations, including the mini-mental state examination (MMSE) and clinical dementia rating (CDR) scale, and underwent *APOE* genotyping. Subjects were questioned to determine the presence/absence of subjective cognitive impairment (SCI). Austin Health Human Research Ethics Committee approved the study and subjects provided written informed consent.

### Image Acquisition

PiB-PET scans were acquired using an Allegro PET camera (Phillips Medical Systems, The Netherlands). A transmission scan was performed for attenuation correction. Participants received 370 MBq ^11^C-PiB over 1 minute, and a 20-minute acquisition (6× 5-minute frames) in 3D-mode was performed beginning 50 minutes after injection. PET images were reconstructed using a 3D RAMLA algorithm [10]. Summed images from the 50 to 70 minute time-frame were used in this study. Sagittal T1-weighted MRI brain scans were acquired at baseline and ∼18 months using a 3D magnetization prepared rapid gradient echo sequence on a Siemens Trio Tim 3T scanner, with 1×1 mm in-plane resolution; 1.2 mm slice thickness; repetition time/echo time/inversion time = 2,300 ms/2.98 ms/900 ms; flip angle 9°; field-of-view 240×256; and 160 slices.

### Image Analysis

#### MRI volumes and atrophy rates

Images were corrected for intensity inhomogeneity using the N3 algorithm [21]. Following 9-degrees-of-freedom registration of the follow-up to baseline scans and differential bias correction [22], whole brain and hippocampal segmentations were produced at each time-point using BrainMAPS [23] and HippoMAPS [24] respectively. In line with our practice for clinical trials, each segmented scan underwent a visual quality control process, with minimal manual editing where necessary. Ventricular volumes were delineated semi-automatically using the MIDAS software package [25]. Volume change over time (millilitres) was obtained using the boundary shift integral (BSI). Volume change was measured for ventricles (VBSI) [26], hippocampus (HBSI) [24] and whole brain (KN-BSI) [27]. Baseline total intracranial volume (TIV) was estimated using the FreeSurfer [28] image analysis suite v4.5.0, which employs the Buckner method [29].

#### Amyloid PET processing

Subsequent to the processing described above, NiftySeg [30] was used to fractionate each MR voxel into grey matter(GM), white matter and CSF tissue classes, and to segment the cerebellum. Each MR scan was then parcellated into regions of interest using the label fusion procedure MultiSTEPS [31] with the Hammers 30-subject atlas [32]. To achieve the best label propagation possible, atlases were non-rigidly registered using NiftyReg [33], first to the group-wise average image, and subsequently to each individual image. PET images were rigidly registered to their corresponding MRI using a block matching approach [34], and the MRI segmentations re-sliced into PET space using trilinear interpolation. Mean PiB uptake values in cerebellar GM (voxels containing ≥95% GM) were used to normalise cerebral uptake, producing Standardized Uptake Value Ratios (SUVRs). Neocortical masks were created by combining a subset (frontal, temporal, parietal and occipital lobes plus insula and cingulate cortex) of the parcellations with the GM segmentations, discarding voxels with fractional volume below 5%. The robust weighted mean SUVR under this mask was calculated using the GM fractional volumes as weights and excluding the highest and lowest 5% SUVR values.

### Statistical Analyses

We used a hierarchical cluster analysis based on Ward’s linkage [35] (using the “cluster wardslinkage” command in Stata) to dichotomise the cohort on the basis of SUVR. Those with higher SUVRs were designated PiB-positive, the remainder PiB-negative. Standard linear regression models were fitted to explore the association between SUVR, baseline brain volumes, atrophy rates and age. Baseline characteristics, cross-sectional brain volume and annualised atrophy rates were compared between PiB-positive/negative groups using two-sample t-tests allowing for unequal variances, Wilcoxon rank-sum tests, and Fisher’s exact test. Further regression models were fitted which allowed for a shift in mean atrophy rate and change in slope at the predefined SUVR cut-off. Data from PiB-positives were used to estimate sample sizes to provide 80% power, 5% type-1 error, to detect 25% absolute reduction in whole brain, ventricular and hippocampal change over 18-months, and to detect 25% absolute reduction as a proportion of PiB-positive, PiB-negative group difference. Bias-corrected and accelerated 95% bootstrap (100,000 samples) confidence intervals were found for sample size estimates. Analyses were performed in Stata12 (Stata Corp, TX).

## Results

### Baseline Characteristics and Rates of Change

Data from 67 individuals were available. Visual inspection of the MRI scans revealed the development of a possible tumour in one individual, who was excluded. Demographic and imaging data for the remaining 66 are shown in Table 1. Mean±SD age was 73.5±7.3 years, MMSE 29.0±1.3, and 35% were *APOE4* positive. Thirteen individuals (19.6%) had SCI; there was a trend for these individuals to have higher MMSE scores than the remainder (29.5±1.0 vs. 28.9±1.3, p = 0.06). Annualized rates of change for the whole group were 5.5±7.0 ml/yr for brain loss, 1.4±1.9 ml/yr for ventricular expansion and 0.031±0.06 ml/yr for total (left+right) hippocampal atrophy.

**Table 1:** Baseline demographics, APOE genotypes, Brain Volumes, and 1-Year Rates of Atrophy for all individuals included in the study.

Characteristic	Mean ± SD, or %	95% CI
Age, yr	73.5±7.3	71.7–75.3
Gender, % male	45.5%	–
APOE ε4, % positive	34.8%	–
MMSE	29.0±1.3	28.7–29.3
CDR-Sum of boxes	0 (n = 64); 0.5 (n = 4)	–
Subjective Cognitive Complaints	n = 13 (19.7%)	–
MRI Scan Interval, days	558±95	534.4–581.3
Baseline brain volume, ml	1088±105	1062–1113
Baseline TIV	1512±159	1473–1550
Baseline ventricular volume, ml	35.4±20.2	30.4–40.1
Baseline left hippocampal volume, ml	2.71±0.37	2.62–2.80
Baseline right hippocampal volume, ml	2.60±0.37	2.51–2.69
Baseline total hippocampal volume, ml	5.31±0.72	5.11–5.48
Inter-scan interval (days)	558±95	534–581
Whole brain atrophy rate, ml/yr	5.46±7.0	3.94–7.24
Ventricular expansion rate, ml/yr	1.39±1.90	0.95–1.86
Left hippocampus loss, ml/yr	0.016±0.037	0.007–0.025
Right hippocampus loss, ml/yr	0.015±0.030	0.008–0.022
Total hippocampal atrophy rate, ml/yr	0.031±0.061	0.016–0.046

NC = normal control; SD = standard deviation; CI = confidence interval; MMSE = Mini Mental State Examination; CDR-SB = Clinical Dementia Rating – Sum of Boxes.

### Correlating Baseline Amyloid Deposition with Baseline Brain Volumes and Rates of Change

**Figure 1:**
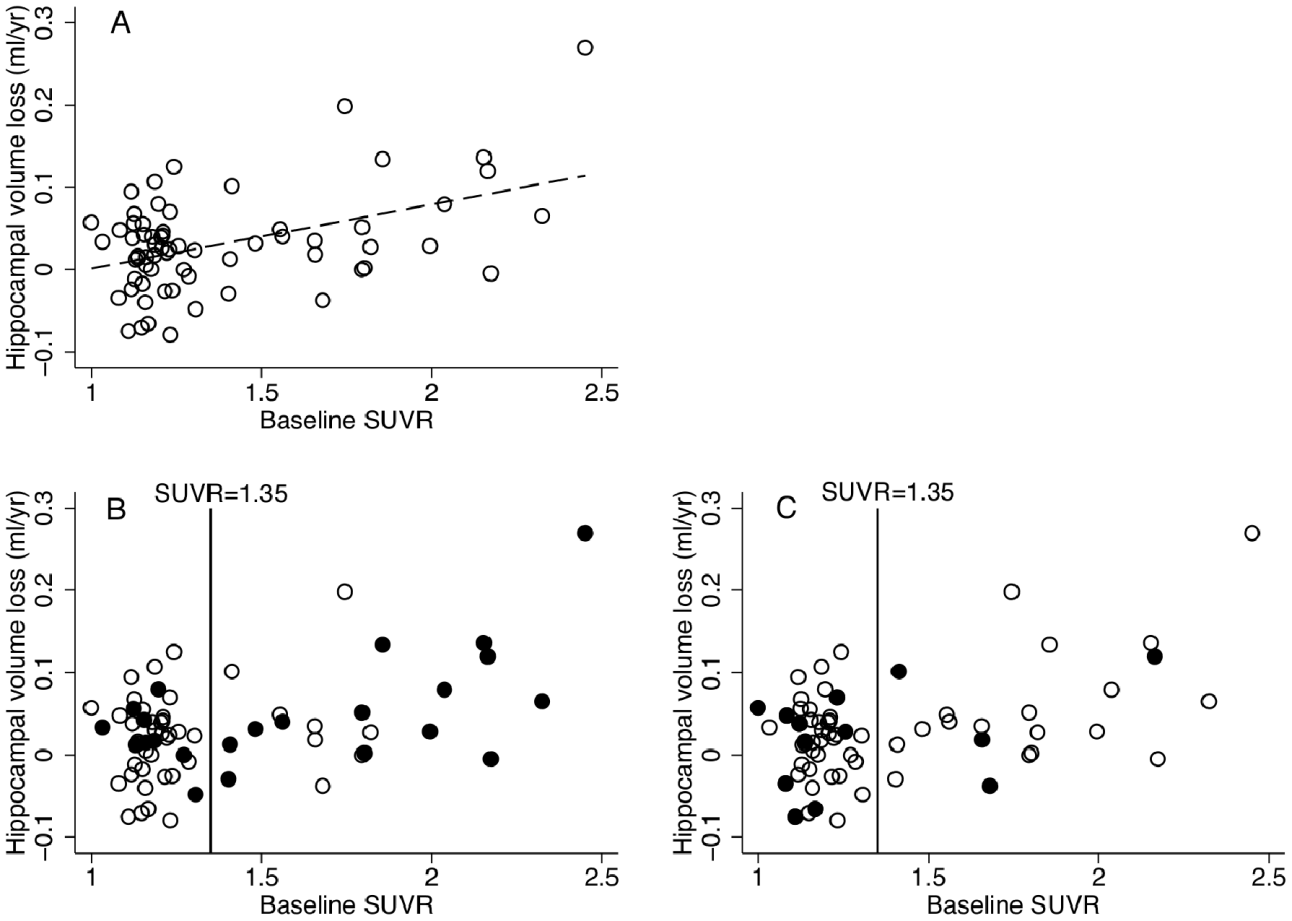
Plots of baseline SUVR vs. annualized hippocampal change. (A) The relationships and regression slope for the group as a whole; (B) APOE4 individuals shown in closed circles; (C) subjective cognitive impairment individuals shown in closed circles. SUVR values between 1.31 and 1.40 separate the groups: a reference line at SUVR = 1.35 is shown for illustration.

There was no significant relationship between baseline SUVR and brain (p = 0.12, R^2^ = 0.02) or ventricular volume (p = 0.34, R^2^ = 0.00), but a significant relationship between (greater) SUVR and (smaller) baseline brain/TIV ratio (p = 0.007, R^2^ = 0.09), and hippocampal volume both with (p = 0.011, R^2^ = 0.08) and without (p = 0.008, R^2^ = 0.09) correction for TIV. Increasing baseline SUVR was associated with higher rate of volume loss in the right hippocampus (p<0.001), left hippocampus (p<0.001), and total hippocampi (p<0.001, R^2^ = 0.19) (Figure 1A). There was borderline evidence for an association between baseline SUVR and ventricular expansion rate (p = 0.07, R^2^ = 0.04), and a non-significant but positive association with whole brain atrophy (p = 0.2, R^2^ = 0.01).

Across the whole cohort, both increasing baseline SUVR (p = 0.003, R^2^ = 0.12) and increasing hippocampal atrophy rate (p = 0.006, R^2^ = 0.10) were associated with increasing age, but we found no statistically significant relationship between age and brain or ventricular rates of change. In a multivariable regression analysis, the relationship between baseline SUVR and baseline hippocampal volume or brain/TIV ratio was not significant when accounting for age and gender. However the relationship between SUVR and hippocampal atrophy rate remained statistically significant after adjusting for age and ApoE4 positivity, with no evidence that this was influenced by either gender or years of education.

### Comparing PiB-positive and PiB-negative Groups

The cluster analysis separated subjects into two distinct groups, one with SUVR≤1.31 (PiB-negative, n = 44), the other with SUVR>1.40 (PiB-positive, n = 22). Baseline SUVR correlated with increasing age in the PiB-negative (p<0.05, R^2^ = 0.07), but not PiB-positive group (p = 0.4), although the difference in slopes was not statistically significant (p = 0.074). There was a significant association between total hippocampal volume loss and baseline SUVR (independent of age) in the PiB-positive (p = 0.02, R^2^ = 0.2) but not PiB-negative group (p = 0.7) (Figure 1B,C), although the difference in slopes failed to reach significance (p = 0.21).

Table 2 shows the baseline and longitudinal results for the PiB-positive and PiB-negative groups. PiB-positive individuals were older (76.8±5.7 vs. 71.8±7.4, p<0.01), and more likely to be *APOE4* positive (63.6% vs. 19.2%, p<0.01) but there were no significant differences in baseline ventricular, or hippocampal volumes (with or without correction for TIV) or TIV, either including or excluding SCI individuals. The PIB-positive group had smaller brain/TIV volumes than the PIB-negative group (70.7±3.7 vs. 72.8±3.3%, p = 0.03), but this was no longer significant when age and gender were accounted for. The PiB-positive group had greater combined hippocampal loss (0.06±0.07 vs. 0.02±0.05 ml/yr, p = 0.02) independent of baseline age or gender. The PiB-positive group also had non-significantly higher rates of whole brain (7.1±9.3 vs. 4.7±5.4 ml/yr, p = 0.3) and ventricular (2.0±3.0 vs. 1.1±0.9 ml/yr; p = 0.2) change. Given the relatively small sample size we repeated the analysis of the hippocampal atrophy rates using non-parametric statistics (Wilcoxon rank-sum test), confirming significantly higher rates of atrophy in the PiB-positive group (median[IQR] = 0.04[0.09] vs. 0.02[0.06] ml/yr, p = 0.04]. At an SUVR cut-off of 1.5– a value used in other studies [10], [13] –18/69 subjects were amyloid-positive; mean hippocampal atrophy rates were hardly changed and still significantly higher than in the amyloid-negative group using both parametric (0.06±0.08 vs. 0.02±0.06 ml/yr, p = 0.04) and non-parametric (0.04[0.1] vs. 0.02[0.06]ml/yr, p = 0.02) approaches, with consistently higher, but non-significant rates of whole brain atrophy and ventricular expansion.

**Table 2:** Baseline demographics, APOE genotypes, Brain Volumes, and 1-Year Rates of Atrophy in PiB-positive and PiB-negative individuals.

Characteristic	NC-low, SUVR ≤1.31, n = 44		NC-high, SUVR >1.4, n = 22		*p*
	Mean ± SD	95% CI	Mean ± SD	95% CI	
Age, yr	71.8±7.4	69.6–74.1	76.8±5.7	74.3–79.4	0.004
Gender, % male	47%	–	50%	–	0.61
APOE ε4, % positive	20.5%	–	63.6%	–	0.001
MMSE	29.1±1.2	28.8–29.5	28.8±1.3	28.2–29.4	0.35
CDR-Sum of boxes = 0.5 (n,%)	2 (4.5%)	–	2 (9.1%)	–	0.60
Scan Interval, days	558.8±102.1	527.8–589.8	555.9±82.4	519.4–592.5	0.90
Baseline brain volume, ml	1098±105	1066–1130	1066±103	1021–1112	0.25
Baseline TIV	1512±162	1463–1561	1511±157	1441–1580	0.98
Baseline ventricular volume, ml	35.5±20.6	29.2–41.7	35.2±19.9	26.4–44.1	0.96
Baseline left hippocampal volume, ml	2.75±0.33	2.65–2.85	2.64±0.43	2.45–2.83	0.28
Baseline right hippocampal volume, ml	2.63±0.33	2.53–2.73	2.54±0.43	2.35–2.73	0.40
Baseline total hippocampal volume, ml	5.38±0.65	5.18–5.57	5.17±0.85	4.80–5.55	0.33
Whole brain atrophy rate, ml/yr	4.7±5.4	3.0–6.3	7.1±9.4	2.9–11.2	0.28
Ventricular expansion rate, ml/yr	1.07±0.88	0.81–1.34	2.01±3.01	0.68–3.35	0.16
Left Hippocampus loss, ml/yr	0.008±0.031	−0.001–0.018	0.032±0.043	0.013–0.051	0.03
Right hippocampus loss, ml/yr	0.008±0.023	0.001–0.016	0.029±0.036	0.013–0.045	0.03
Total Hippocampal atrophy rate, ml/yr	0.016±0.047	0.004–0.031	0.060±0.075	0.027–0.093	0.02

NC = normal control; SD = standard deviation; CI = confidence interval; MMSE = Mini Mental State Examination; CDR-SB = Clinical Dementia Rating – Sum of Boxes.

### Excluding Individuals with Subjective Cognitive Impairment or CDR = 0.5

Excluding individuals with SCI (n = 13) did not materially alter results: there was still a significant relationship between SUVR and hippocampal atrophy rate (p<0.001, R^2^ = 0.2) with weak evidence for associations with ventricular expansion (p = 0.08) and whole brain atrophy rate (p = 0.1); and significantly higher rates of hippocampal atrophy in the PiB-positive group (p = 0.04). Whilst all subjects’ clinical diagnoses were assessed by a clinical review panel, to ensure that the four subjects with CDR = 0.5 were not influencing the results we re-ran our analyses excluding these individuals. Across the group as a whole there was still a significant relationship between SUVR and hippocampal atrophy rate (p = 0.02); the relationship with ventricular expansion was now significant (p = 0.04), and with whole brain atrophy rate borderline significant (p = 0.07). Comparing the PiB-positive and PiB negative groups, again there were significantly higher rates of hippocampal atrophy in the former (p = 0.04).

### Sample Size Calculations

Recruiting amyloid-positive subjects to power a one-year treatment trial to detect 25% absolute slowing of cerebral atrophy rates (equivalent to ∼35% slowing if the maximum effect was to reduce atrophy rate to the mean loss in the PiB-negative group) requires 442 (95% CI 180,1650) subjects/arm using whole brain atrophy, 542 (180,1649) using ventricular expansion and 384 (195,1080) using hippocampal atrophy rates.

## Discussion

In this study we demonstrate a significant relationship between hippocampal atrophy rates and amyloid load in cognitively normal individuals, and show that this is independent of increasing age. In the absence of significant demographic or baseline cross-sectional volumetric differences, and independent of including/excluding individuals with SCI, PiB-positive controls (∼1/3 of the cohort) had significantly higher rates of hippocampal atrophy. Finally, we provide sample size estimates for therapeutic trials seeking to assess disease-modification effects using rates of hippocampal (and brain) atrophy as outcome measures in asymptomatic amyloid-positive individuals who may be at risk of AD.

As a group, the rates of volume change over time are very similar to those we have previously derived using similar techniques in a larger (n = 199, mean age ∼76 yrs) cohort of ADNI controls [26]. Here rates of whole brain atrophy were 5.6±6.9 ml/yr, and in ADNI 6.3±6.1 ml/yr; total hippocampal atrophy 0.04±0.1 vs. 0.05±0.1 ml/yr.; and ventricular expansion 1.4±1.9 vs. 1.4±1.6 ml/yr. Despite preferential recruitment of SCI individuals in AIBL, these results suggest that the two groups are comparable; and with the caveats that rates of *APOE4* positivity are high (∼29%, ADNI; ∼35%, AIBL), and that individuals recruited to observational studies may not represent the population in general, these results may be reasonably generalizable to populations who might be recruited for prevention trials.

We found a relationship between baseline amyloid load and rates of atrophy, significantly so for hippocampal volume loss. This result – based on volumetric measures of change using well validated techniques – confirm those previously shown in AIBL using voxel-wise analyses [36]; and again, despite differences in inter-scan interval and recruitment strategy are very similar to the relationships we have previously reported in ADNI controls, dichotomised using CSF Aβ1-42 [37]. Whilst previous work has shown a relationship between age and baseline SUVR, [10] we found that the relationship between brain atrophy rate and SUVR for the group as a whole was independent of age, gender or baseline MMSE, providing further evidence for a relationship between amyloid deposition and neurodegeneration, as has been previously demonstrated in very early AD [38]. Whilst there was a significant relationship between increasing amyloid load and both smaller hippocampal volumes and brain/TIV ratios across the group as a whole, these relationships were no longer present once age and gender were accounted for. Whilst numbers are small, taken together these findings are consistent with the formulation that amyloid deposition leads to excess hippocampal atrophy rates which, over time, result in differences in mean hippocampal volumes at a group level.

In keeping with previous amyloid PET studies, we dichotomised the group into amyloid-positive/negative on the basis of SUVR, determining a cut-off consistent with a value of 1.4 defined in previous AIBL studies [36], [39]. At this level, ∼1/3 of this cognitively normal cohort were amyloid-positive, in keeping with previous PET [10], [36], [39] and CSF [9], [40] studies. As in prior studies, PiB-positive individuals were older and more likely to be *APOE4* positive than PiB-negatives [10]. Whilst hippocampal volumes were slightly smaller in the PiB-positive group, there were no significant differences in baseline hippocampal volumes between the groups, either including or excluding subjects with SCI, or correcting for TIV. These findings, similar to those we reported when comparing normal controls dichotomised on the basis of CSF Aβ1-42, [36] are at odds with some studies reporting lower temporal lobe volumes in PiB-positive individuals [11], [13], and others including Chetelat *et* *al* who found increased temporal lobe (including hippocampal) volume in PiB positive, SCI-negative AIBL controls [39]. It is unclear whether these discrepancies relate to methodological differences, relatively small sample sizes, or the more limited and specific regions we assessed. Importantly, only 3/22 of the amyloid-positive group had total hippocampal volumes outside the lower limit of the 95% reference range for the amyloid-negative group, suggesting that the vast majority would fulfil proposed criteria for isolated asymptomatic Aβ-amyloidosis [7]. Whilst we found no differences in ventricular volumes between the groups, the PIB positive group had significantly (3%, p = 0.03) smaller brain volume as a proportion of TIV. However, this difference was no longer significant (p = 0.075) once age and gender were accounted for.

Comparing longitudinal rates of atrophy with the PiB-negative group, PiB-positives had ∼50% higher rates of brain atrophy, ∼double the rate of ventricular expansion, and ∼three-fold – and significantly – higher rates of hippocampal atrophy. These differences are remarkably similar to those we have previously shown in ADNI control subjects [36], an independent sample dichotomised using a different biomarker of amyloid deposition, CSF Aβ1-42 which is inversely correlated to PET amyloid load [41] (Table 3). Importantly, dichotomising the groups at a slightly higher SUVR of 1.5, as has been used in some AIBL [10], [11], [17] and other studies [42] produced very similar results. These results provide evidence for a fundamental difference between healthy, amyloid-negative, aging where rates of atrophy in the mid-70 s are ∼0.3–0.4%/year for brain and ∼0.4–0.6%/yr for combined hippocampi, and higher rates of loss in asymptomatic (CSF or PIB positive) amyloidosis of ∼0.7–0.9%/yr for brain and ∼1.3–1.4%/yr for hippocampi. It is notable that in asymptomatic amyloidosis, rates are not only intermediate between “normality” and those seen in mild cognitive impairment, but also show a disproportionate effect on the hippocampi relative to whole brain, in keeping with the hypothesis that these individuals might be in the earliest stages of AD [43].

**Table 3:** Baseline demographics, APOE genotype, neuropsychometry, SUVR, brain volumes and annualised rates of atrophy in amyloid positive and negative normal controls from this study compared to ADNI amyloid positive/negative individuals defined on the basis of CSF Aβ1-42.

Characteristic	Amyloid “negative” Mean ± SD		Amyloid “positive” Mean ± SD	
	AIBL (n = 44)	ADNI (n = 65)	AIBL (n = 22)	ADNI (n = 40)
Age, yr	71.8±7.4	74.9±5.1	76.8±5.7	76.3±5.1
Gender, % male	47%	51%	50%	55%
APOE ε4, % positive	20.5%	10.8%	63.6%	47.5%
MMSE	29.1±1.2	29.0±1.1	28.8±1.3	29.2±0.9
Baseline brain volume, ml	1098±105	1054±103	1066±103	1077±105
Baseline ventricular volume, ml	35.5±20.6	35.8±20.9	35.2±19.9	39.4±16.2
Baseline total hippocampal volume, ml	5.38±0.65	5.26±0.70	5.17±0.85	5.17±0.62
Whole brain atrophy rate, ml/yr	4.7±5.4	4.4±5.3	7.1±9.4	9.3±6.9
Ventricular expansion rate, ml/yr	1.07±0.88	0.95±1.14	2.01±3.01	2.04±1.93
Hippocampal atrophy rate, ml/yr	0.02±0.05	0.03±0.09	0.06±0.08	0.07±0.10

NC = normal control; SD = standard deviation; CI = confidence interval; MMSE = Mini Mental State Examination.

We found a significant relationship between amyloid load and hippocampal atrophy rate in the amyloid-positive but not amyloid-negative group. Although, perhaps due to limited power, differences between slopes did not reach significance, this is consistent with the hypothesis that the relationship between amyloid accumulation and atrophy differs between normal aging and early AD; and that amyloid burden may need to reach a critical level before excess neurodegeneration is triggered [6]. It is noteworthy that individuals with SCI had rather higher MMSE scores than the remainder of the group and, as shown in Figure 1 (C), were rather more likely to be PiB-negative than positive. Whilst based on a relatively small sample size, these results provide some preliminary data to suggest that for trials aiming to recruit PiB-positive controls, there may be more to be gained through enriching for *APOE4* which is strongly, albeit not exclusively, associated with PiB-positivity [44] (Figure 1 B), than through enrichment for SCI. However, as is evident from the Figure 1, amyloid positivity is not exclusive to *APOE4* individuals, and so whilst preferentially recruiting *APOE4* individuals is likely to increase the numbers of amyloid-positive individuals detected, this strategy comes with the risk of potentially reducing the generalizability of any results to the wider population. Whilst numbers are small and there was no evidence for differences in rate of hippocampal atrophy between *APOE4* carriers (n = 14, 0.067±0.078 ml/yr) and non-carriers (n = 8, 0.049±0.072 ml/yr, p = 0.59) within the amyloid-positive group, potential differential effects of therapy based on *APOE4* status as has been observed in immunotherapy studies [45] may also influence recruitment strategy.

For pre-symptomatic prevention clinical trials, these data suggest that using hippocampal atrophy rate, ∼380 PiB-positive individuals would be required per arm of an 18-month placebo/controlled study to detect 25% absolute slowing of brain atrophy (i.e. ∼35% decline relative to “healthy”, amyloid-negative, normal aging). It is worth noting that these numbers refer to the numbers needed to complete the study with adequate imaging at baseline and follow-up, and in practice trial design must allow for drop-outs and poor scans. We report sample sizes over 18-months, the approximate inter-scan interval in this study. It is likely that within-subject SDs (and hence sample sizes) would be slightly higher if measured over one year, and slightly lower over longer intervals [46]; nonetheless, these figures are not dissimilar to those we have previously estimated based on one-year ADNI data [37]. Whilst the wide confidence intervals should be noted, sample sizes of this order are within the scale of proposed prevention studies, such as the A4 study which proposes to recruit ∼1000 amyloid-positive individuals [19]. Prevention studies are likely to be run over relatively long periods and to use conversion status or cognitive scores as principal outcome measures. In this context, rates of hippocampal or brain atrophy may be particularly valuable in providing interim assessments of disease modification [47].

There are a number of caveats of this study, particularly with reference to applications in therapeutic studies. The number of individuals included is modest, only two scanning time-points were used for analysis, and our estimates are based on a 25% absolute slowing of atrophy: accordingly any sample size estimates need to be viewed with caution particularly given the wide confidence limits (195–1080) we report. Similarly, the relatively small number of individuals in the study may also explain some of the differences in sample size estimates between this study and our prior report [37]. Some of the hippocampal volumes in this study appear to show increases over time (i.e. negative rates of atrophy). This may reflect real physiological changes (e.g. an individual’s level of hydration), or image artefacts (e.g. motion), noise or voxel drift in the MR scans, which may result in apparent negative changes when the underlying ground-truth atrophy rate is close to zero [48]. Finally, whilst the rationale for using atrophy as an outcome is that a disease-modifying drug would slow rates of change, prior amyloid vaccination studies in AD [49] and emerging results from some recently completed trials [50] in AD have shown unexpected excess volume loss in patients on active treatment, demonstrating that there may be an unexpected dissociation between biomarker changes and clinical outcomes. It may however be that the effects of treatment on those already in the clinical stages of AD might differ from those at an early, pre-symptomatic, stage of AD pathology, which is the target group for our study; and it is likely that biomarker changes may provide invaluable mechanistic information even in failed studies. For these reasons, it is vitally important to have an understanding of the relationship between atrophy and amyloid load in asymptomatic amyloidosis. Further larger studies with longer follow-up are required to explore these issues in more detail, but our results suggest that there does appear already to be a “signal” of excess atrophy in the asymptomatic amyloidosis group, which could be exploited for the purposes of clinical trials.

In conclusion we provide further evidence that asymptomatic amyloidosis is not a benign state, but is associated with increased neurodegeneration. Longitudinal follow-up is required to determine whether the combination of amyloidosis and increased rates of cerebral atrophy can predict accurately whether an individual will develop AD, and if so over what time frame. These results suggest however that hippocampal and brain atrophy, measured using validated, quantitative techniques, may be a useful outcome measure, potentially providing additional information in trials designed to detect disease-modification in asymptomatic individuals with amyloidosis.
